# Artificial intelligence (AI) in nursing administration: Challenges and opportunities

**DOI:** 10.1371/journal.pone.0319588

**Published:** 2025-04-01

**Authors:** Omar Qaladi, Mukhlid Alshammari, Abdullah Abdulrahim Almalki

**Affiliations:** 1 Community and Psychiatric Mental Health Nursing Department, College of Nursing, King Saud University, Riyadh, Saudi Arabia; 2 Nursing Department, College of Applied Medical Science, University of Hafr Albatin, Hafr Albatin, Saudi Arabia; 3 Ministry of Health, Taif, Saudi Arabia; University of Hafr Al-Batin, SAUDI ARABIA

## Abstract

Artificial Intelligence (AI) is increasingly transforming nursing administration by enhancing operational efficiency and supporting data-driven decision-making. This study explores registered nurses perceptions of AI in Saudi Arabia, focusing on both challenges and opportunities. A cross-sectional survey of 202 nurses revealed that 93.6% believe AI improves understanding, and 88.1% feel it enhances the quality of learning. Significant correlations were found between years of experience and AI usage (r =  0.342, p <  0.001) and between sources of information and AI perception (r =  0.146, p =  0.039). While 80.7% expressed concern that AI could reduce critical thinking, 76.8% feared job displacement. These findings underscore the need for training, ethical guidelines, and support systems to foster effective AI integration, enhancing nursing practice while addressing concerns around professional roles.

## Introduction

Technological advancements in AI are transforming healthcare by improving diagnostic accuracy, decision-making, and administrative efficiency [1,2]. Nursing benefits from AI’s potential to optimize patient care and streamline operations, yet requires new competencies in technology and data use [3,4]. Globally, AI has enhanced workforce management and decision-making in nursing administration, though challenges remain around ethics, critical thinking, and empathy [3,4], underscoring the need for targeted training and support.

In recent years, artificial intelligence (AI) has transformed nursing administration globally, enhancing operational efficiency and supporting data-driven decision-making. For instance, AI-based predictive analytics tools assist nurse managers in optimizing staffing and resource allocation by forecasting peak demand periods based on patient admissions and acuity levels, reducing nurse workload and improving patient care outcomes [5]. Additionally, AI-driven workflow management systems automate administrative tasks such as documentation, billing, and inventory management, which traditionally consume a significant portion of nurses’ time [6]. By employing Natural Language Processing (NLP) tools to streamline documentation processes, AI allows nurses to spend less time on paperwork and more on direct patient care [7].

Furthermore, data analytics tools powered by AI are enabling nursing administrators to make informed decisions through the analysis of large datasets. These tools identify trends in patient satisfaction, clinical outcomes, and operational performance, which guide quality improvement initiatives and optimize healthcare delivery [8,9]. Such examples underscore the potential of AI to reshape nursing administration globally, providing a solid foundation for exploring the unique challenges and opportunities associated with integrating AI in nursing practice.

Understanding nurses’ attitudes and behaviors toward AI applications is essential for successful implementation in clinical practice and for identifying future training needs. A review study, revealed that nurse practitioners have positively shifted their views on AI, seeing it as a valuable tool for analyzing data, recognizing patterns, and supporting diagnoses. However, concerns remain regarding the reduction of human intervention in patient care, job security, and ethical issues related to using AI in sensitive healthcare contexts [4,8]. Furthermore, a study among nurses in Germany, found that while nurses recognize AI’s potential to reduce administrative burdens and enhance monitoring, few feel confident in their AI knowledge, expressing concerns about impacts on patient interaction and job security [10].

Research on AI integration in nursing practice in the Middle East is limited. In Qatar, a study found that most healthcare students had a positive attitude towards AI, finding it useful and reliable for accelerating work processes and making diagnoses, despite concerns about job displacement and lack of empathetic care [11]. Similar positive attitudes were found among doctors and medical students in Syria though concerns about job reduction and the need for training persisted [12]. These findings support earlier recommendations for AI integration in medical care [8,13]. However, in Saudi Arabia, limited research has been conducted on AI’s specific role and impact within nursing administration, leaving a notable gap in understanding how AI might influence nursing roles, job security, and overall healthcare quality in this context. By addressing these global concerns and focusing on Saudi Arabia, this study aims to bridge the gap in understanding AI’s integration in nursing administration and to provide contextually relevant insights that could support AI adoption strategies suited to the region.

## Methods

### Study design and aims

This research adopted a cross-sectional design to capture a snapshot of nurses’ perceptions of AI integration in nursing administration and practice. The cross-sectional approach facilitated the collection of data at a single point in time, offering insights into the prevailing attitudes and characteristics of the study population [14].

### Setting and sample

The study was conducted in two referrals hospitals: King Abdulazize Spcialist Hospital (KASH) and King Faisal Medical Complex (KFMC) in Saudi Arabia. The inclusion criteria comprised nurses currently working in these hospitals, and participation is voluntary. A simple random sampling technique was employed to ensure representation of all ages, both genders, across different employment levels. All nurses who agreed to participate in the study and met the sample requirements.

### Recruitment and data collection

Data were collected using convenience sampling from two referrals hospitals, King Abdulazize Spcialist Hospital (KASH) and King Faisal Medical Complex (KFMC) in Saudi Arabia. Data were collected from 1^st^ April to 31^st^ May 2024. The sample size was calculated using this formula n =  z2p (1-p)/e2 (n =  sample size, z =  degree of confidence based on standard normal distribution, p =  roughly proportion of the population that shows the trait, and e =  allowed margin of error [14,15]. To achieve statistical reliability, a confidence level of 95% was selected, with a margin of error set at 5%, aligning with similar studies targeting professional perceptions. The final sample size of 148 accounted for a 10% increase to mitigate non-responses and incomplete surveys. This approach ensured comprehensive representation while maintaining statistical power. Participants were selected based on specific criteria, including licensure in Saudi Arabia, at least one year of inpatient experience, and current roles in nursing administration. These criteria ensured a sample of experienced nurses with relevant backgrounds, aligning with the study aim to assess AI perceptions in nursing practice. The questionnaire was administered in English, as it is the formal communication language in Saudi hospitals. No translation was required, and participants did not report language barriers during the study.

### Measurements

A structured questionnaire was developed to gather quantitative data on participants’ socio-demographic characteristics, AI usage, familiarity and challenges with AI, and perceptions related to AI in administration and nursing practice. The survey instrument underwent a rigorous validation process, involving expert review and pilot testing to ensure clarity and relevance.

#### Demographic information.

Participants provided information on age, gender, Level of education, Working field, Years of nursing experience, and the source of information regarding AI. These demographic variables were crucial for analysing the correlation with perceptions.

#### AI usage and familiarity.

Participants were queried on their utilization of AI technology in education. The survey assessed their familiarity with specific AI tools commonly used in nursing administration and practice and their perceived difficulty in using high-profile AI technology.

#### Perceptions in nursing administration and nursing practice.

The questionnaire explored participants’ perceptions of AI in education, focusing on its role in providing efficient learning experiences, improving understanding, and enhancing the overall level of education. In the context of nursing administration and practice, perceptions were gauged regarding AI’s potential in fast and accurate diagnosis, improving healthcare measures, and assisting rather than replacing critical thinking skills.

#### Validity and reliability of the instrument.

The questionnaire was reviewed by an expert panel to evaluate whether the questions agreed with the scope of the items, identify the extent to which these items reflect the concepts of the research problem and to judge whether the instrument is statistically valid, and that the questionnaire is sufficiently well designed to provide relationships between the examined variables. Reliability was measured through calculating Cronbach’s alpha coefficient for the total questionnaire and the six subdomains.

#### Pilot study.

A pilot study was conducted on 10% of the gathered sample to test reliability and applicability of the study to ascertain the feasibility, applicability, and clarity of the tool, and modifications were incorporated. nurses in the pilot study were excluded from the study.

### Data analysis

SPSS (IBM, v 27.0) was used for data analysis. Descriptive statistics, including frequencies and percentages, was employed to summarize participants’ socio-demographic characteristics and responses to survey items. Pearson correlation coefficients was calculated to explore correlations between socio-demographic variables and participants’ attitudes towards AI.

### Ethical considerations

This study complied with the Declaration of Helsinki and was approved by the ethics committee in King Abdulaziz City for Science and Technology (KACST) IRB (no. H-02-T-123) (Taif Health Cluster), Saudi Arabia. Informed consent was obtained from all study participants. Completion and return of the questionnaire by the participants indicated their consent to participate in the study as explained in the participation information sheet. Participants were informed that they were free to withdraw from the study at any time and that anonymity and confidentiality would be maintained through not using personal identifiers or reporting potentially identifiable information. All data were anonymized at the point of collection by excluding identifiers that could link responses to individuals. Data were securely stored on encrypted devices with access limited to the primary investigator, reducing the risk of potential breaches. In light of the sensitive nature of AI and its implications on job security, these measures were emphasized to participants to alleviate any concerns of reprisal based on their responses

## Results

### Demographic characteristics

A total of 202 nurses participated in the study as shown in Table 1. The majority (44.6%) of respondents are aged between 23-26 age group, with a higher representation of females (61.9%). Regarding years of experience, 35.1% have 4-6 years of experience, while 32.2% have more than 10 years. Notably, 42.6% of participants received information on AI through mass media, while 14.9% obtained information from professionals.

**Table 1:** Socio-demographic variables.

Variables	Frequency *N* = 202	Percentage (%)
**Age in years**		
23–26	90	44.6
27–30	62	30.7
31–35	42	20.8
More than 35	08	4.0
**Gender**		
Male	77	38.1
Female	125	61.9
**Years of experience**		
1–3 years	43	21.3
4–6 years	71	35.1
7–10 years	23	11.4
More than 10 years	65	32.2
**Source of information received on AI**		
Mass media	86	42.6
Friends	41	20.3
Professionals	31	15.3
Health care Sittings	30	14.9
Others	14	6.9

### AI usage and familiarity

The study explores nurses’ perceptions of general aspects of AI technology in nursing as shown in Table 2. A majority (82.7%) of respondents are familiar with specific AI tools commonly used in nursing, indicating a broad awareness and integration of AI technologies in their practice. However, 84.6% feel that using high-profile AI technology is difficult, suggesting that complexity and usability remain significant barriers. In terms of safety and reliability, 74.7% believe that AI is safe and reliable, highlighting some reservations. Additionally, 88.1% agree that AI reduces emotional exhaustion or physical limitations, suggesting that AI can positively impact nurse well-being. These findings underscore the importance of improving AI usability and training for nurses to fully leverage AI technologies. While there is general confidence in AI’s safety and its potential to alleviate workload, addressing usability concerns is crucial for broader acceptance and effective implementation.

**Table 2:** General aspects of artificial intelligence (AI) technology.

Statements	Strongly Agree		Agree		Neutral		Disagree		Strongly Disagree	
	*N*	%	*N*	%	*N*	%	*N*	%	*N*	%
I am familiar with specific Artificial Intelligence (AI) tools commonly used in nursing.	98	(48.5)	69	(34.2)	2	(1.0)	19	(9.4)	14	(6.9)
I feel it’s difficult to use high profile AI technology	120	(59.4)	51	(25.2)	1	(0.5)	18	(8.9)	12	(5.9)
I feel the AI is safe and reliable	94	(46.5)	57	(28.2)	22	(10.9)	12	(5.9)	12	(5.9)
AI reduces emotional exhaustion or physical limitation	126	(62.4)	52	(25.7)	10	(5.0)	2	(1.0)	12	(5.9)

### Perceptions in education and nursing practice

The study reveals positive perceptions of AI among nurses in education, nursing practice, communication, and evaluation as illustered in Table 3. In education, 91.1% of respondents agree that AI helps provide efficient learning experiences, and 93.6% believe AI improves the level of understanding. Additionally, 88.1% feel that combining AI with education will enhance learning quality.

**Table 3:** Level of perception of nurses on artificial intelligence (AI) and its role in nursing administration.

Statements	Strongly Agree		Agree		Neutral		Disagree		Strongly Disagree	
	*N*	%	*N*	%	*N*	%	*N*	%	*N*	%
**Education/ Teaching** I feel Artificial Intelligence (AI) helps to provide efficient learning experiences for the students.	121	(59.9)	63	(31.2)	2	(1.0)	6	(3.0)	10	(5.0)
Artificial Intelligence (AI) will help to improve level of understanding	115	(56.9)	74	(36.6)	11	(5.4)	1	(0.5)	1	(0.5)
I feel level of education will improve, if it is combined with Artificial Intelligence (AI) Technology	126	(62.4)	52	(25.7)	15	(7.4)	3	(1.5)	6	(3.0)
Use of Artificial Intelligence (AI) will enhance quality of work, productivity and my performance level	90	(44.6)	82	(40.6)	15	(7.4)	1	(0.5)	14	(6.9)
**Nursing Practice** Artificial intelligence comprises many health care technologies that transforms nurses’ role and enhance to provide more personalized patient care.	74	(36.6)	82	(40.6)	44	(21.8)	2	(1.0)	0	(0)
AI tools helps in improving all levels of healthcare measures	98	(48.5)	59	(29.2)	44	(21.8)	1	(0.5)	0	(0)
Artificial Intelligence (AI) Technology will help in fast and accurate diagnosis among the patients	104	(51.5)	94	(46.5)	3	(1.5)	1	(0.5)	0	(0)
AI tools are intended to assist and not replace the critical thinking skills of the nurses	112	(55.4)	64	(31.7)	21	(10.4)	5	(2.5)	0	(0)
The Ministry of Health seeks to use artificial intelligence technologies to improve the health sector and make it better in the world by collecting data and using it to support decision-making and develop services provided to citizens	106	(52.5)	49	(24.3)	32	(15.8)	0	(0)	15	(7.4)
**Communication** I feel use of Artificial Intelligence (AI) will help to improve easy and accurate communication among healthcare team members.	113	(55.9)	53	(26.2)	15	(7.4)	11	(5.4)	10	(5.0)
**Evaluation** I feel AI based Evaluation process will reduce subjectivity	104	(51.5)	55	(27.2)	25	(12.4)	1	(0.5)	17	(8.4)
I feel AI can be used for creating test and examinations	99	(49.0)	52	(25.7)	7	(3.5)	10	(5.0)	34	(16.8)

In nursing practice, 77.2% agree that AI technologies enhance personalized patient care, while 77.7% believe AI tools improve healthcare measures. Furthermore, 98% think AI aids in fast and accurate diagnosis, and 87.1% see AI as an assistant, not a replacement for critical thinking skills.

Regarding communication, 82.1% agree that AI improves communication among healthcare teams. In evaluation, 78.7% feel AI-based processes reduce subjectivity, and 74.7% believe AI can be used for creating tests and examinations. These findings indicate strong support for AI’s role in enhancing education, clinical practice, communication, and evaluation within nursing, highlighting the perceived benefits of AI integration.

### Challenges and concerns

The study identifies several significant challenges associated with the use of AI in nursing as shown in Table 4. A substantial 81.7% of respondents are concerned that technical malfunctions of AI could lead to negative outcomes, highlighting reliability issues. Furthermore, 80.7% believe that AI reduces critical thinking and decision-making skills among nurses. Emotional assessment by AI is also a concern, with 91% agreeing that AI struggles to evaluate patients’ emotional well-being effectively. A lack of knowledge and confidence in using AI is seen as a challenge by 71.3% of participants, indicating the need for better training. Job security is another major issue, with 76.8% fearing AI could result in job losses. Ethical concerns are raised by 71.3%, who worry about breaches in academic integrity. Additionally, 62.8% cite the high cost of AI as a significant disadvantage. Lastly, 76.8% believe that reliance on AI could marginalize healthcare providers’ roles. These findings underscore the need for robust education, ethical guidelines, and support systems for AI integration in nursing.

**Table 4:** Challenges of artificial intelligence (AI) applications.

Statements	Strongly Agree		Agree		Neutral		Disagree		Strongly Disagree	
	*N*	%	*N*	%	*N*	%	*N*	%	*N*	%
Technical malfunction of AI could results in a negative outcome	124	(61.4)	41	(20.3)	23	(11.4)	1	(0.5)	13	(6.4)
The use of AI reduces critical thinking, judgement and decision-making skill among nurses.	133	(65.8)	30	(14.9)	29	(14.4)	2	(1.0)	8	(4.0)
AI has low ability to assess the emotional well-being of the patient	112	(55.4)	72	(35.6)	1	(0.5)	8	(4.0)	9	(4.5)
Lack of knowledge and confident in using AI become a challenge	117	(57.9)	27	(13.4)	40	(19.8)	7	(3.5)	11	(5.4)
AI may results in the loss of traditional job opportunities among the nurses	110	(54.5)	45	(22.3)	18	(8.9)	8	(4.0)	21	(10.4)
Breach of academic integrity and Ethical issue may be a concern.	96	(47.5)	48	(23.8)	28	(13.9)	12	(5.9)	18	(8.9)
The high cost is one of the disadvantages of using AI technology	115	(56.9)	12	(5.9)	47	(23.3)	2	(1.0)	26	(12.9)
Total reliance on technology in care may marginalize the role of health care providers or reduce job opportunities	105	52.0	50	24.8	21	10.4	17	8.4	9	4.5

### Correlation analysis

The study explored the correlations between socio-demographic variables and nurses’ perceptions of AI as illustrated in Table 5. The significant positive correlation between years of experience and favorable AI perception (r =  0.342, p <  0.001) suggests that experienced nurses may perceive AI as a complementary asset, potentially aiding their clinical expertise rather than substituting it. Additionally, nurses receiving information through professional sources tend to hold more supportive views on AI integration (r =  0.146, p =  0.039), emphasizing the influence of reliable information sources in shaping perceptions. Targeted communication and professional training on AI can thus positively impact its acceptance, underscoring the need for trusted, informed discussions surrounding AI’s role in healthcare.

**Table 5:** Correlation between socio-demographic variables and the level of perception of nurses on artificial intelligence (AI) and its role in nursing administration.

Variables	Pearson Correlation Coefficient (r)	Level of Significance (P value)
Gender	−.083	.242
Years of experience:	.342^**^	000^***^
Source of Information	.146^*^	039^*^
Use of AI	.205^**^	.003^**^
Familiar with (AI) tools used in nursing.	.217^**^	002^**^
Difficult to use high profile AI	−.081	.250
AI is safe and reliable	−.167^*^	.017
AI reduces emotional exhaustion or physical limitation	.117	.099
**Education/ Teaching** AI helps to provide efficient learning experiences for the students.	.021	.768
AI will help to improve level of understanding	.228^**^	001^**^
level of education will improve, it is combined with Artificial Intelligence (AI) Technology	.020	.782
AI will enhance quality of work, productivity and my performance level	.178^*^	.011^*^
**Nursing Practice** Artificial intelligence comprises many health care technologies	−.001	.990
AI tools help in improving all levels of healthcare measures	.047	.509
AI Technology will help in fast and accurate diagnosis	.063	.370
AI tools are intended to assist and not replace the critical thinking skills of the nurses	.149^*^	.034^*^
The Ministry of Health seeks to use artificial intelligence technologies to improve the health sector	.2623^**^	.000^***^
**Communication** Use of AI will help to improve easy and accurate communication	−.086	.221
**Evaluation** AI based Evaluation process will reduce subjectivity	.146	.039
AI can be used for creating test and examinations	.475^**^	.000^**^
**Challenges of AI Application** technical malfunction in AI could results in a negative outcome	0.018	.804
AI reduces critical thinking, judgement and decision-making skill among nurses.	.076	.284
AI has low ability to assess the emotional well-being of the patient	.057	.421
Lack of knowledge and confident in using AI become a challenge	.072	.306
AI may result in the loss of traditional job opportunities among the nurses	.144^*^	.040^*^
Breach of academic integrity and Ethical issue may be a concern.	.150^*^	.033^*^
The high cost is one of the disadvantages of using AI technology	.210^**^	003^**^
Total reliance on technology in care may marginalize the role of health care providers or reduce job opportunities	−.067	.390

* Correlation is significant at the 0.05 level (2-tailed),

** Correlation is significant at the 0.01 level (2-tailed),

*** Correlation is significant at the 0.001 level (2-tailed).

Familiarity with AI tools was positively correlated with perceptions of AI’s benefits in education and nursing practice, such as improving understanding and enhancing work performance. However, there were also concerns about AI’s safety and reliability, with some nurses feeling uncertain about these aspects. The correlations suggest that individual characteristics such as age, gender, and experience significantly influence how nurses view AI. These insights are valuable for educators and policymakers to tailor AI integration strategies to meet the specific needs and expectations of diverse nursing populations, thereby facilitating smoother and more effective AI adoption in healthcare settings.

## Discussion

The study reveals nurses’ nuanced perceptions of AI, recognizing both its benefits and challenges. The high familiarity with AI tools among participants suggests increasing integration of AI in nursing, aligning with studies from Egypt and Germany that indicate positive trends in AI acceptance among nurses [10,16]. These findings echo global patterns in healthcare technology adoption, seen in countries such as the United States and the United Kingdom [8,13]. However, the perceived difficulty in using high-profile AI technologies underscores the need for comprehensive training programs to improve usability and confidence among nurses. The findings are in agreement with studies that found nurse practitioners support the integration of AI by providing courses on AI fundamentals, ethics, and practical applications [10,17]. Both perspectives point to the importance of continuous learning and institutional support, ensuring healthcare staff can adapt to evolving AI technologies.

The positive perceptions of AI’s impact on education, with a majority believing it enhances learning experiences and understanding, align with the broader literature on AI’s potential to revolutionize educational paradigms in healthcare [18,19]. Furthermore, a recent study shows how targeted training can improve caregiving outcomes, suggesting that integrating AI with similar educational initiatives could further enhance patient care [17]. This indicates the need to integrate AI into the global nursing education system [20]. Additionally, the view that AI can improve work quality and productivity indicates an acknowledgment of its role in enhancing clinical efficiency. This aligns with previous study, who found that AI not only optimizes workloads but also strengthens communication among staff and with patients, contributing to better patient care and reduced burnout [3].

Despite these positive attitudes, significant concerns remain. The perception that AI reduces critical thinking and decision-making skills highlights a fear of over-reliance on technology, which could undermine essential nursing competencies [4]. The concern about AI’s inability to assess patients’ emotional well-being reflects an important limitation, emphasizing the irreplaceable value of human empathy in nursing care [9,21]. Furthermore, the fear of job displacement due to AI, as indicated by a significant portion of the respondents, reflects global concerns about technology replacing human roles in healthcare [14]. This aligns with findings from a study in the UAE, where nurses viewed AI as a career threat [22]. Ethical concerns, including breaches of academic integrity and the high cost of AI implementation, further complicate the acceptance and integration of AI in nursing [8].

Moreover, the correlation results of this study confirm previous findings regarding the complex relationship between socio-demographic variables and perceptions of AI [7,8,23]. The results of this study indicate a significant correlation between years of experience and the use of AI, as well as between sources of information and AI perception. These findings align with Buchanan et al. [8] and Teng et al. [24], who also observed that personal and professional backgrounds profoundly influence engagement with AI technologies. For instance, more experienced nurses may have more practical insights, resulting in varied levels of acceptance or resistance compared to their less experienced counterparts, as noted by Chan & Zary [7].

Recent studies in Middle Eastern healthcare settings provide a broader context to our findings on AI perceptions among Saudi nurses. For instance, research from Qatar and Syria reveals a generally positive attitude toward AI’s potential, although concerns about job displacement persist [12,25]. Furthermore, a recent study underscores the vital role of the nurse-patient relationship, especially in caring for older adults, emphasizing that AI should support rather than replace human aspects of care [26]. These findings align with our study, where participants acknowledged AI’s benefits but also voiced apprehensions about its impact on job security. Situating our findings within this regional context underscores the shared concerns and optimism surrounding AI integration in healthcare across neighboring Middle Eastern countries.

Additionally, nurses who receive information about AI through professional channels tend to have a more nuanced and supportive view of AI integration, supporting the studies conducted by Ronquillo et al. [5] and Secinaro et al. [4]. These correlations highlight the importance of targeted communication and training strategies that consider the diverse backgrounds of nursing staff, ensuring that AI adoption is inclusive and effective across different demographic segments. Tailoring AI training and implementation programs to address specific needs and concerns of different nurse groups can lead to more successful integration of AI in healthcare settings, as suggested by Robert [6] and Jha et al. [9].

Understanding these demographic influences can help healthcare institutions develop more effective AI adoption strategies, enhancing technological proficiency while preserving essential human elements in nursing care. This approach aligns with the recommendations by Abuzaid et al. [22] and Swed et al. [12], emphasizing the need for balanced AI integration that supports both technological and human aspects of healthcare.

### Strength and limitations of the study

This study provides valuable insights into the perceptions of AI among nurses in Saudi Arabia, marking the first study of its kind in this context. Its strengths include the comprehensive exploration of both positive perceptions and concerns. However, the study has some limitations. Being conducted in only two referral hospitals limits the generalizability of the results to other healthcare settings in Saudi Arabia. The cross-sectional design captures perceptions at a single point in time, which may not reflect changes over time. Additionally, the reliance on self-reported data can introduce response bias [14]. Despite these limitations, this pioneering study lays the groundwork for future research and provides critical insights that can inform the integration of AI in nursing practice in Saudi Arabia.

## Conclusion

This study shows that nurses generally view AI positively, recognizing its potential to enhance education, improve efficiency, and aid in diagnosis. However, concerns remain around AI’s impact on critical thinking, emotional assessment, job security, and ethical issues. Socio-demographic factors, such as experience and sources of information, influence these perceptions, underscoring the need for targeted training and communication. Effective AI integration requires training programs that familiarize nurses with AI in patient care and data management, as well as data protection policies to ensure privacy. Future research should include longitudinal studies to track changes in nurses’ perceptions as AI use becomes routine, providing insights into its impact on critical thinking, job security, and nursing roles.

## Abbreviations

AI: Artificial intelligence
KACST: King Abdulaziz City for Science and Technology
KASH: King Abdulazize Spcialist Hospital
KFMC: King Faisal Medical Complex.
